# Probiotic Insights from the Genomic Exploration of *Lacticaseibacillus paracasei* Strains Isolated from Fermented Palm Sap

**DOI:** 10.3390/foods13111773

**Published:** 2024-06-05

**Authors:** Phoomjai Sornsenee, Komwit Surachat, Dae-Kyung Kang, Remylin Mendoza, Chonticha Romyasamit

**Affiliations:** 1 Department of Family and Preventive Medicine, Faculty of Medicine, Prince of Songkla University, Songkhla 90110, Thailand; ezipnary@gmail.com; 2 Department of Biomedical Sciences and Biomedical Engineering, Faculty of Medicine, Prince of Songkla University, Songkhla 90110, Thailand; komwit.s@psu.ac.th; 3 Department of Animal Biotechnology, Dankook University, Cheonan 31116, Republic of Korea; dkkang@dankook.ac.kr (D.-K.K.); emilyn.mendoza@gmail.com (R.M.); 4 Department of Medical Technology, School of Allied Health Sciences, Walailak University, Nakhon Si Thammarat 80160, Thailand; 5 Center of Excellence in Innovation of Essential Oil and Bioactive Compounds, Walailak University, Nakhon Si Thammarat 80160, Thailand

**Keywords:** probiotic, comparative genome analysis, bacteriocin, antimicrobial activity, lactic acid bacteria

## Abstract

This study focused on *L. paracasei* strains isolated from fermented palm sap in southern Thailand that exhibit potential probiotic characteristics, including antibiotic susceptibility, resistance to gastrointestinal stresses, and antimicrobial activity against various pathogens. However, a thorough investigation of the whole genome sequences of *L. paracasei* isolates is required to ensure their safety and probiotic properties for human applications. This study aimed to sequence the genome of *L. paracasei* isolated from fermented palm sap, to assess its safety profile, and to conduct a comprehensive comparative genomic analysis with other *Lacticaseibacillus* species. The genome sizes of the seven *L. paracasei* strains ranged from 3,070,747 bp to 3,131,129 bp, with a GC content between 46.11% and 46.17% supporting their classification as nomadic lactobacilli. In addition, the minimal presence of cloud genes and a significant number of core genes suggest a high degree of relatedness among the strains. Meanwhile, phylogenetic analysis of core genes revealed that the strains possessed distinct genes and were grouped into two distinct clades. Genomic analysis revealed key genes associated with probiotic functions, such as those involved in gastrointestinal, oxidative stress resistance, vitamin synthesis, and biofilm disruption. This study is consistent with previous studies that used whole-genome sequencing and bioinformatics to assess the safety and potential benefits of probiotics in various food fermentation processes. Our findings provide valuable insights into the potential use of seven *L. paracasei* strains isolated from fermented palm sap as probiotic and postbiotic candidates in functional foods and pharmaceuticals.

## 1. Introduction

Lactic acid bacteria (LAB) are gram-positive bacteria that are non-spore-producing, cocci or rods, catalase-negative, fastidious, tolerant to low pH, and have low G + C content [1]. The genomes of LAB are distinguished by their compact size, which varies from 1.23 Mb (*Lactobacillus sanfranciscensis*) to 4.91 Mb (*L. parakefiri*) [2]. They typically produce lactic acid as the major metabolic end product [3]. They are found in the guts of humans and animals and are common in fermented food and drink products, such as yogurt, kefir, cheese, sauerkraut, pickles, and fermented palm sap [4,5,6]. LAB genera include *Lactococcus*, *Pediococcus*, *Streptococcus*, *Aerococcus*, *Vagococcus*, *Lacticaseibacillus* (*Lactobacillus*), *Dolosigranulum*, *Alloiococcus*, *Carnobacterium*, *Leuconostoc*, *Enterococcus*, *Oenococcus*, *Tetragenococcus*, and *Weissella* [1,5]. LAB are generally recognized as safe (GRAS) and have been given Qualified Presumption of Safety (QPS) status by the European Food Safety Authority (EFSA) [7]. A previous study showed many of the beneficial effects of lactic acid bacteria. They can improve skin conditions and prevent skin diseases [8]. *Lactobacillus* strains inhibited *Neisseria gonorrhoea* and *Candida albicans* [9]. *E. faecalis* inhibit toxigenic *C. difficile* [10]. The LAB strain (LBbb0141) contained an antimicrobial compound with a wide spectrum and was inhibitory to Gram-positive and Gram-negative strains [11]. Moreover, the *Lacticaseibacillus paracasei* strain PS23, isolated from the feces of healthy humans, has anti-inflammatory effects [12].

*Lacticaseibacillus paracasei* (previously named *Lactobacillus paracasei*) [13] has been studied and isolated from many sources, especially fermented food and drink products. *L. paracasei* is composed of the closely related species *L. casei* and *L. rhamnosus*, among others [14].

It is also used as a starter culture for dairy products in the food industry and as probiotics [4,5]. Recently, we isolated *L. paracasei* strains from fermented palm sap collected in southern Thailand. Although it is not the predominant species in fermented palm sap, it may contribute to its health-promoting properties. These isolates met the criteria to qualify as probiotic, including antibiotic susceptibility, resistance to the gastrointestinal environment, and adherence to human intestinal cells. They exhibited antimicrobial activity against various pathogens [5], which is an important characteristic of probiotics. Moreover, the lyophilized cell-free supernatants (LCFSs) of these isolates significantly reduced biofilm formation and eradicated established biofilms. LCFSs contain antioxidant compounds (phenolic and flavonoid) and showed antioxidant and anti-inflammatory activities in RAW 264.7 cell lines [15]. *L. paracasei* T0901 was considered a highly acceptable component in a probiotic–banana rehydrated beverage [16]. These results indicate that *L. paracasei* isolated from fermented palm sap are promising probiotic and postbiotic candidates that can be used in functional foods and pharmaceuticals. However, before these *L. paracasei* isolates are considered safe for human applications and are attributed with probiotic properties, a thorough investigation of the entire genome sequence is required. Moreover, Onwuakor et al. [17] found that *L. paracasei* J23 had antibacterial activity against *Salmonella typhimurium* by using bacteriocin. Many advantages of *L. paracasei* strains have been reported, including antimicrobial and antibiofilm activity, immune system stimulation, stress modulation, anti-inflammatory, anti-obesity, and antioxidative properties, and improvements in intestinal bacterial microbiota [18,19]. 

Genome sequencing has revolutionized the ways in which the biology, physiology, ecology, evolution, and applications of organisms are studied. Currently, the National Center for Biotechnology Information (NCBI) database has around 95,511 genomes of organisms classified under the order Lactobacillales. Out of these, 12,259 genomes (1639 of which are complete) belong to the *Lactobacillaceae* family [14]. Previous studies have used whole-genome sequencing (WGS) technologies and bioinformatics to investigate the safety and potential benefits of probiotics used in food fermentation, such as fermented pork sausages [20,21], fermented milk [22,23], and fermented congee [24]. These studies conducted an in silico safety assessment using the complete nucleotide sequence of the bacterial genome to confirm safety and unveil traits derived from the predicted genes. However, a significant gap persists in the literature concerning the genomic exploration of probiotics. There is an evident need for a comprehensive genomic analysis of *L. paracasei* strains.

This study aimed to sequence the genome of *L. paracasei* strains isolated from fermented palm sap, evaluate their safety profiles, and perform a comprehensive comparative genomic analysis with other *Lacticaseibacillus* species. These efforts are aimed at offering valuable insights into the possible applications of *L. paracasei* strains as potential candidates for probiotics and postbiotics in functional foods and pharmaceuticals.

## 2. Materials and methods

### 2.1. Bacterial Strains, Culture Conditions, and DNA Isolation

Seven *L. paracasei* strains (T0601, T0602, T0901, T0902, T1301, T1304, and T1901) were previously isolated from fermented palm sap [5]. A single colony of each *L. paracasei* isolate was cultivated in Man, Rogosa, and Sharpe (MRS) broth (HiMedia, Mumbai, India) at 37 °C for 24 h under anaerobic conditions.

Genomic DNA was purified and extracted using a DNeasy extraction kit (QIAGEN, Hilden, Germany) following the manufacturer’s instructions. Briefly, the bacterial cell was suspended in 180 μL of lysis buffer and incubated for 30 min at 37 °C. Then, 25 μL of proteinase K was mixed in 200 μL of buffer AL and incubated at 56 °C for 30 min. Ethanol (200 μL) was added to the DNA sample, which was then centrifuged for 1 min at 610× *g*, washed with 500 μL of buffer AW2, and eluted with buffer AE. The purity of the DNA was estimated using a spectrophotometer by measuring the absorbance at 260 and 280 nm (A260/A280) and via agarose gel electrophoresis.

### 2.2. Genome Assembly and Annotation

DNA specimens were sent to the Beijing Genomics Institute for short-read WGS using 150 bp paired-end reads on the MGISEQ-2000 platform. Subsequently, the sequencing reads were assembled and annotated using the comprehensive BacSeq v1.0 pipeline [25]. BacSeq integrates multiple bioinformatics pipelines, including SPAdes [26], Prokka [27], QUAST [28], and BUSCO [29], to assemble, annotate, and assess the quality and completeness of genome assemblies. Mobile genetic elements, prophages, and antimicrobial resistance genes (ARGs) were assessed using mobileOG-db [30], Phigaro [31], and VirulenceFinder [32] and ResFinder web-based tools [33], respectively. For the ARG search, the criteria were a threshold of 90% and a minimum length of 60%. CRISPR (clustered regularly interspaced short palindromic repeats) arrays and the corresponding Cas proteins were identified using CRISPRCasFinder [34]. Ribosomally synthesized and post-translationally modified peptides and genes encoding bacteriocin-encoding genes were identified via sequence similarity search using the BAGEL4 webserver [35].

### 2.3. Pangenome Analysis and Comparative Genomics

Seven genomes of *L. paracasei* were used for a comprehensive comparative analysis and pan-genome evaluation. The Roary pipeline [36] was used to examine the pan-genome, using a 95% BLASTp threshold and standard parameters to identify core, accessory, and unique protein families. Subsequently, multiple gene alignments and phylogenetic trees were generated using Geneious [37] and the neighbor-joining method. Bootstrap testing was conducted with 500 repetitions to assess tree reliability. Additionally, a comparative analysis of *L. paracasei* genomes was performed using Proksee [38] and BLASTn [39] to determine coding sequence similarities, and OrthoANI [40] was used for average nucleotide identity (ANI) analysis.

## 3. Results and Discussion

### 3.1. Genome Features and Stability of the L. paracasei Strains

**Figure 1:**
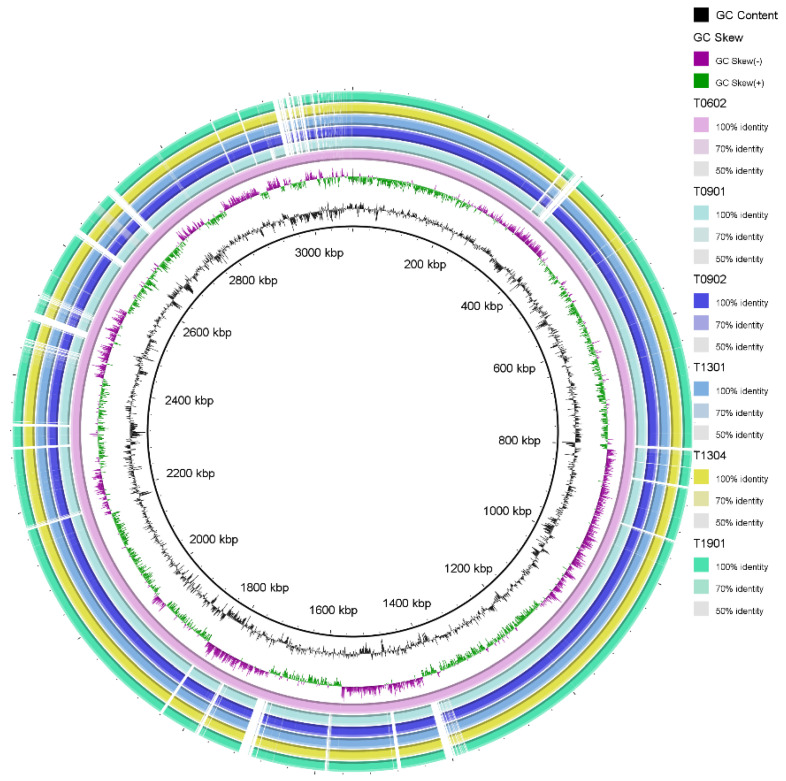
Blast Ring Image Generator (BRIG) diagram showing the concatenated sequences of *Lacticaseibacillus paracasei* strains with the genome of strain T0601 as a reference. The two inner circles represent the GC content (black) and GC skew (violet and green).

Seven *L. paracasei* strains, namely T0601, T0602, T0901, T0902, T1301, T1304, and T1901, isolated from fermented palm sap [5] were examined in this study. The genomic characteristics of these strains are summarized in Table 1 and Figure 1. Their genomes had a total length ranging from 3,070,747 to 3,131,129 bp, with T1301 having the largest size (3,131,129 bp) and T0602 having the smallest (3,070,747 bp). The GC content across the strains ranged from 46.11% to 46.17%.

Genome annotation revealed various genetic elements. The number of coding sequences (CDS) varied from 2918 to 3021, with T0902 having the highest number (3021) and T1304 having the lowest (2918). All strains consistently had 3–4 rRNA and 56 tRNA genes. In addition, each strain contained a single copy of the tmRNA gene. Genome metrics of LAB strains can serve as approximate indicators of their lifestyle. Previous reports showed that the genome size of lactobacilli can vary from 1.28 to 4 Mb, depending on their specific environmental niche preferences [41,42]. Throughout the evolutionary process, some species of LAB, such as *Lactobacillus sensu lato*, have undergone genome reduction, particularly during the transition from free-living to nomadic and matrix-associated bacteria. In contrast, free-living and nomadic strains that encounter diverse environments tend to possess larger genomes, ranging from 3 to 4 Mb, to support their survival. Among these strains, *L. paracasei* belongs to the *L. casei* group along with the nomadic species *L. casei* and *L. rhamnosus.* These species exhibit genomes with a median length of approximately 2.9 Mb and a GC content ranging from 46% to 47%. They primarily inhabit similar niches, such as dairy products, but can also establish associations with host organisms [19,43]. In this study, the genome sizes of the seven *L. paracasei* strains provided evidence to support their classification as nomadic lactobacilli. The genome size and gene number of these strains were similar to those of *L. paracasei* DTA93 isolated from healthy infant feces (3.02 Mb, 2990 genes), with very similar GC contents (46.2%) [44]. However, all seven *L. paracasei* strains showed larger genome sizes and higher gene numbers than *L. paracasei* SD1 isolated from the human oral cavity (2.99 Mb, 2984 genes) [23]. The observed differences in genome size and gene number among strains may indicate their adaptation to distinct environmental niches. None of the strains contained intact phages: T0601 had two, T0902 and T1901 had four, and T0602, T0901, T1301, and T1304 had three incomplete phages. In addition, T0901, T1301, and T1304 harbored one questionable phage (Table 1). Almost all *L. paracasei* strains carried four prophage regions, except T0601 and T0602, which carried three prophage regions (Supplementary Table S1). Prophages are commonly found in probiotic strains used in dairy fermentation, including *Lactococcus*, *Bifidobacterium*, and *Lactobacillus* [45,46]. According to Ventura et al. [45], the genome of a single strain of *Lactiplantibacillus plantarum* contains at least four prophage-like entities. Prophages are frequently detected in *Lactobacillus* strains, typically ranging from 1 to 5 prophages per genome [46]. This suggests that prophages are prevalent in the genomes of probiotic bacteria and emphasizes their significance in the context of dairy fermentation and related applications. Multiple predicted intact prophage regions within the same strain also showed variations in structural composition. For *L. paracasei* BL23, previous studies have reported the presence of five intact prophages in strains [47]. *L. paracasei* strain EG9, isolated from cheese, contains 15 prophages [48]. Our finding of three intact prophages within the genome supports the existing body of evidence and is consistent with that of previous studies, which have also reported the presence of intact prophages in *Lactobacillus* strains. The identification of these intact prophages in our study strengthens their significance in the genetic composition and diversity of the studied *Lactobacillus* strains. *L. paracasei* strains have a well-established record of safe consumption, and extensive research has demonstrated their excellent tolerability when administered in the form of supplements or incorporated into fermented food products. Previous studies have consistently reported positive outcomes regarding the safety and tolerability of *L. paracasei* strains, supporting their suitability as dietary supplements and fermented foods [49].

### 3.2. Comparative Genomics and Pangenome Analysis

**Figure 2:**
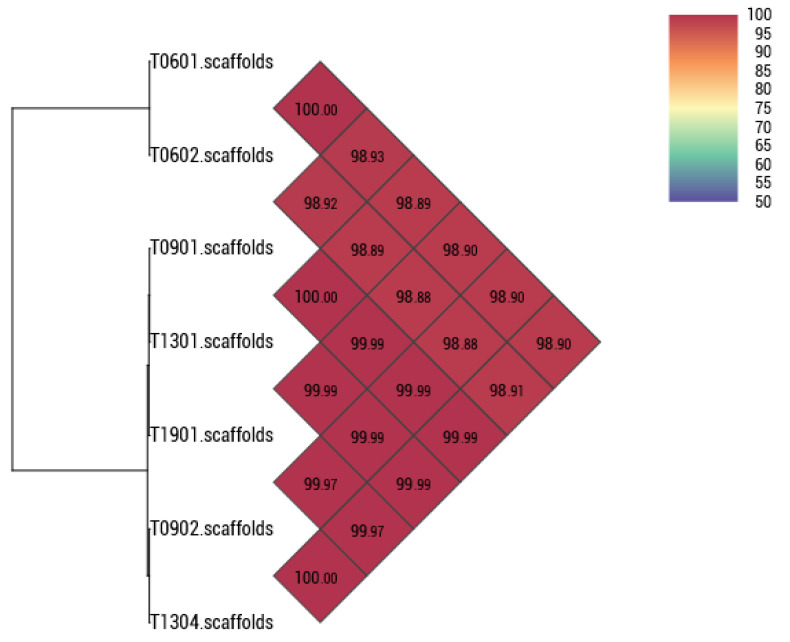
Heatmap of OrthoANI values of the seven *Lacticaseibacillus paracasei* strains calculated using the OAT software (Version 0.93.1).

The degree of genomic similarity of the seven strains with closely related species was calculated using OAT software (Version 0.93.1) [50]. The OrthoANI value among the closely related species (Figure 2) was 99.88% (T0602 and T0901, T0602 and T0902). *L. paracasei* T0601 was compared with the related *L. paracasei* T0602, with a value of 100%. 

**Figure 3:**
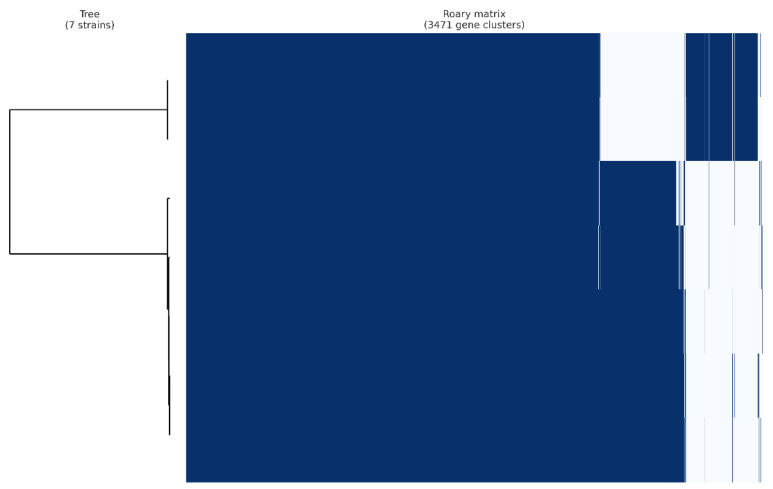
Gene presence/absence matrix from pangenome analysis of 7 *Lacticaseibacillus paracasei* strains using the Roary pipeline. Each row shows the gene profile of each isolate.

**Figure 4:**
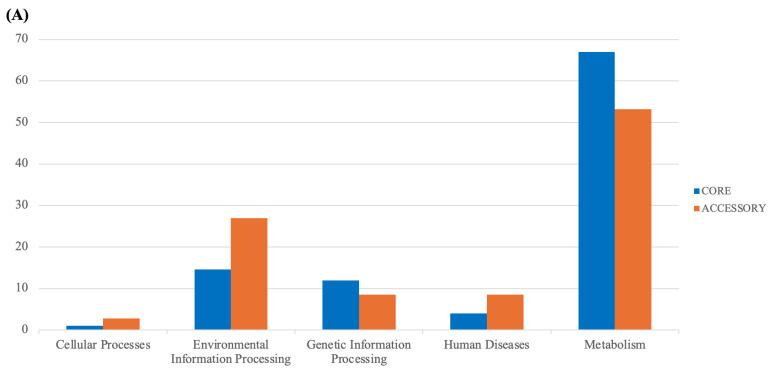

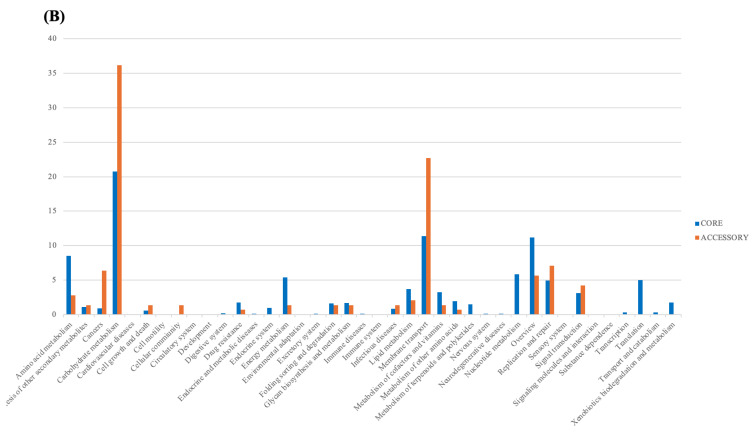
KEGG distribution of *Lacticaseibacillus paracasei* pangenome. (**A**) General and (**B**) detailed distribution.

An analysis of seven *L. paracasei* genomes using Roary revealed that, of a total of 3471 genes, 2478 were identified as core genes, with no soft-core genes detected. In addition, 960 genes were categorized as shell genes and 33 were classified as cloud genes. The minimal presence of cloud genes and the significant number of core genes suggest a high degree of relatedness among the strains studied, as these strains were isolated from fermented palm sap. The consistency in gene content suggests a strong evolutionary connection among the strains, likely originating from their adjustment to the same ecological niche [51,52]. Because they occupy the same environment, these strains may have undergone a relatively recent divergence, preserving the similar functional traits essential for their survival in this environment [53]. However, despite this similarity, phylogenetic analysis based on core genes from the seven strains revealed that these bacteria possessed distinct genes and were grouped into two distinct clades, as illustrated in Figure 3. Strain-specific genes were present in their genomes, many of which encode hypothetical proteins (Figure 4). Notably, only T0602 lacked any distinctive genes in its genome, whereas T0601, T0901, T0902, T1301, T1304, and T1901 possessed 4, 7, 3, 3, 5, and 10 unique genes, respectively. This finding aligns with the results of ANI analysis, which indicated that T0601 and T0602 were closely related, with ANI values exceeding 99%. Similarly, the other five strains that were grouped into separate clades had ANI values of approximately 99%. This finding underscores the genetic variability within *L. paracasei* populations, despite their overall genomic similarity, suggesting a potential adaptation to specific subenvironments within fermented palm sap or ongoing evolutionary processes.

### 3.3. Identification of Genes Related to Probiotic Features

WGS and comparative analyses of the seven *L. paracasei* isolates revealed the presence of multiple genes associated with probiotic functions including gastrointestinal survival, oxidative stress survival, acid, bile salt, temperature, and osmotic shock tolerance, cell wall formation, biofilm formation, vitamin synthesis, and bacteriocin production (Table 2). All six isolates harbored eight genes related to gastrointestinal survival [54]. These genes encode proteins that are crucial for maintaining the structural integrity of the bacteria, enabling them to withstand conditions similar to those observed in the gastrointestinal tract. Eight genes, namely seven *atp*- (A–B, D–H) and *nhaK_*2-encoding acid tolerance proteins, were identified (Table 2). These genes are largely responsible for the assembly and operation of the F0-F1 ATPase proton pump, which maintains cytoplasmic pH by exporting protons following ATP hydrolysis [55]. This system is essential for the survival of bacteria in the acidic environment of the stomach [56]. In addition, the isolates possessed *murE* and *mleS* for bile salt tolerance and *cspB*, *cspLA*, *csp*, *hrcA*, *dnaJ*, *dnaK*, *clpC_1*, and *clpB* for temperature tolerance, which may contribute to their survival and functionality under varying conditions within the host [57]. These six isolates contained a repertoire of genes associated with traits beneficial for probiotic functions, such as genes linked to gastrointestinal survival (*pbpB*, *penA*, *pbpE*, *ponA*, *pbpF_1*, *pbpF_2*, *pbpX*, and *pbp*, which encode penicillin-binding proteins). These proteins play a vital role in the formation and maintenance of the cell wall, thus providing structural integrity to the bacteria against the harsh conditions of the gastrointestinal tract. This genetic makeup supports the phenotypic survival rates of the isolates in highly acidic environments (pH 2 and 3) and in the presence of digestive enzymes such as pepsin and pancreatin [5,55]. Furthermore, osmotic shock tolerance genes, including *grpE*, *gbuA*, *gbuC*, and *gbuB*, may confer resilience against osmotic stress during food processing or within the gut, where osmotic conditions vary. The six isolates harbored an effective oxidative stress response system (Table 2) that supported survival and damage repair under aerobic conditions during production. The correlation between genetic determinants and phenotypic properties, such as hydrophobicity and adhesion to human intestinal cells, may be mediated by genes related to cell wall formation and biofilm formation (*luxS*, *ywqC*, *desR*, and *ccpA_2*), potentially providing a competitive edge for the colonization of the gut environment [58]. The strong ability of a probiotic strain to attach to the gut enhances its persistence in the gut, prevents pathogens, and allows it to interact with the host to protect epithelial cells or modulate the immune system.

The identified genes for vitamin synthesis, which include various *btuD* variants [59] and genes for bacteriocin production (*Thermophilin_A*, LSEI_2386, Sactipeptides, and Thermophilin 13 Chain A), were consistent with the beneficial probiotic functions of the strains. LSEI_2386 peptide is a class IId bacteriocin that exhibits antimicrobial activity against several pathogens [60]. Thermophilin 13 is a broad-host-range antimicrobial substance [61].

## 4. Conclusions

This investigation of the genomes of *L. paracasei* strains isolated from fermented palm sap revealed extensive genomic traits that demonstrate their potential as probiotics. We identified unique genetic elements that contribute to robustness against gastrointestinal and environmental stresses, which are essential for effective probiotic functions. Additionally, comparative genomics highlighted evolutionary adaptations that may favor their use in health-related applications. These genomic insights will pave the way for the further exploration of these strains in clinical settings to confirm their efficacy and safety as next-generation probiotics in functional foods and pharmaceutical formulations.

## Figures and Tables

**Table 1:** Main genome features of *Lacticaseibacillus paracasei* strains.

Feature	T0601	T0602	T0901	T0902	T1301	T1304	T1901
Total length	3,072,098	3,070,747	3,085,678	3,131,129	3,129,100	3,129,120	3,126,709
GC (%)	46.14	46.14	46.17	46.11	46.11	46.11	46.11
N50	158,110	146,425	194,163	166,913	166,913	152,444	166,913
L50	5	6	5	6	6	7	6
Number of contigs	61	60	61	75	78	79	93
CDS	2921	2918	2969	3009	3009	3006	2999
rRNA	3	3	4	4	3	4	4
tRNA	56	56	56	56	56	56	56
tmRNA	1	1	1	1	1	1	1

**Table 2:** The multiple genes associated with the probiotic functions of seven *Lacticaseibacillus paracasei* strains.

Function	Gene	T0601	T0602	T0901	T0902	T1301	T1304	T1901
**Gastrointestinal tract survival**	*pbpB*	+	+	+	+	+	+	+
	*penA*	+	+	+	+	+	+	+
	*pbpE*	+	+	+	+	+	+	+
	*ponA*	+	+	+	+	+	+	+
	*pbpF_1*	+	+	+	+	+	+	+
	*pbpF_2*	+	+	+	+	+	+	+
	*pbpX*	+	+	+	+	+	+	+
	*pbp*	+	+	+	+	+	+	+
**Acid tolerance**	*nhaK_2*	+	+	+	+	+	+	+
	*atpA*	+	+	+	+	+	+	+
	*atpF*	+	+	+	+	+	+	+
	*atpG*	+	+	+	+	+	+	+
	*atpB*	+	+	+	+	+	+	+
	*atpD*	+	+	+	+	+	+	+
	*atpH*	+	+	+	+	+	+	+
	*atpE*	+	+	+	+	+	+	+
**Bile salt tolerance**	*murE*	+	+	+	+	+	+	+
	*mleS*	+	+	+	+	+	+	+
**Temperature tolerance**	*cspB*	+	+	+	+	+	+	+
	*cspLA*	+	+	+	+	+	+	+
	*csp*	+	+	+	+	+	+	+
	*hrcA*	+	+	+	+	+	+	+
	*dnaJ*	+	+	+	+	+	+	+
	*dnaK*	+	+	+	+	+	+	+
	*clpC_1*	+	+	+	+	+	+	+
	*clpB*	+	+	+	+	+	+	+
**Osmotic shock tolerance**	*grpE*	+	+	+	+	+	+	+
	*gbuA*	+	+	+	+	+	+	+
	*gbuC*	+	+	+	+	+	+	+
	*gbuB*	+	+	+	+	+	+	+
	*opuCD*	+	+	+	+	+	+	+
	*opuCC*	+	+	+	+	+	+	+
**Oxidative stress survival**	*hslO*	+	+	+	+	+	+	+
	*nox_2*	+	+	+	+	+	+	+
	*nox_1*	+	+	+	+	+	+	+
	*tpx*	+	+	+	+	+	+	+
	*npr*	+	+	+	+	+	+	+
**Cell wall formation**	*murA1*	+	+	+	+	+	+	+
	*epsH_2*	+	+	+	+	+	+	+
	*ykoT_1*	+	+	+	+	+	+	+
	*tagE*	+	+	+	+	+	+	+
	*dltC*	+	+	+	+	+	+	+
	*dltA*	+	+	+	+	+	+	+
	*dltD*	+	+	+	+	+	+	+
	*dltC*	+	+	+	+	+	+	+
**Biofilm formation**	*ywqC*	+	+	+	+	+	+	+
	*luxS*	+	+	+	+	+	+	+
	*desR*	+	+	+	+	+	+	+
	*ccpA_2*	+	+	+	+	+	+	+
	*brpA_2*	+	+	+	+	+	+	+
	*brpA_4*	+	+	+	+	+	+	+
	*brpA_3*	+	+	+	+	+	+	+
**Vitamin synthesis**	*btuD_14*	+	+	+	+	+	+	+
	*btuD_14*	+	+	+	+	+	+	+
	*btuD_2*	+	+	+	+	+	+	+
	*btuD_8*	+	+	+	+	+	+	+
	*btuD_13*	+	+	+	+	+	+	+
	*btuD_4*	+	+	+	+	+	+	+
	*btuD_15*	+	+	+	+	+	+	+
	*btuD_5*	+	+	+	+	+	+	+
	*btuD_9*	+	+	+	+	+	+	+
	*btuD_12*	+	+	+	+	+	+	+
	*btuD_11*	+	+	+	+	+	+	+
	*btuD_7*	+	+	+	+	+	+	+
	*btuD_6*	+	+	+	+	+	+	+
	*btuD_1*	+	+	+	+	+	+	+
	*btuD_3*	+	+	+	+	+	+	+
**Bacteriocin**	Thermophilin_A	+	+	+	+	+	−	+
	Sactipeptides	+	−	+	+	+	+	+
	LSEI_2386	+	+	+	+	+	+	+
	Thermophilin 13 Chain A	−	−	−	−	−	+	−

## Data Availability

Data are included/referenced in the article or Supplementary Materials.

## Supplementary Materials

The following supporting information can be downloaded at: https://www.mdpi.com/article/10.3390/foods13111773/s1, Table S1: Predicted prophage regions in the genome of *Lacticaseibacillus paracasei* strains.
