# Supplementary material for: Probiotic Insights from the Genomic Exploration of Lacticaseibacillus paracasei Strains Isolated from Fermented Palm Sap
Source: Foods. 2024 Jun 5;13(11):1773. doi: 10.3390/foods13111773 (PMC11172291; doi:10.3390/foods13111773)
Supplement: Supplementary file 1 [file foods-13-01773-s001.zip › foods-3026957-supplementary.pdf]

Supplementary Table S1. Predicted prophage regions in the genome of *Lacticaseibacillus paracasei strains.*

Contig	Completeness	Score	Total Proteins	Region Position	Most Common Phage	GC (%)
L. paracasei T0601						
NODE_1_length_417502_cov_120.331015	incomplete	50	30	332667-364642	PHAGE_Lactob_T25_NC_048625(13)	42.48%
NODE_4_length_271218_cov_128.427814	incomplete	60	46	227228-270121	PHAGE_Lactob_BH1_NC_048737(18)	44.31%
L. paracasei T0602						
NODE_1_length_417499_cov_126.992078	incomplete	50	30	52863-84838	PHAGE_Lactob_T25_NC_048625(13)	42.47%
NODE_3_length_271217_cov_134.280910	incomplete	60	46	227227-270120	PHAGE_Lactob_BH1_NC_048737(18)	44.31%
L. paracasei T0602						
NODE_2_length_137747_cov_247.656723	incomplete	10	10	79243-87714 info_outline	PHAGE_Nodula_vB_NspS_kac65v151_NC_048756(2)	0.5123
NODE_3_length_132037_cov_186.274235	intact	150	47	90384-129768 info_outline	PHAGE_Lactob_LF1_NC_019486(16)	44.95%
L. paracasei T0901						

Contig	Completeness	Score	Total Proteins	Region Position	Most Common Phage	GC (%)
NODE_1_length_437545_cov_154.117295	incomplete	10	9	386566-391945	PHAGE_Bacill_G_NC_023719(2)	49.20%
NODE_3_length_307816_cov_127.929648	incomplete	30	18	47496-65362	PHAGE_Staphy_SPbeta_like_NC_029119(3)	46.89%
NODE_4_length_235316_cov_125.443460	incomplete	20	8	201421-209815	PHAGE_Prochl_P_SSM2_NC_006883(3)	49.51
NODE_3_length_132037_cov_186.274235	intact	150	47	90384-129768 info_outline	PHAGE_Lactob_LF1_NC_019486(16)	44.95%
NODE_8_length_136053_cov_132.574234	questionable	70	16	66279-80812	PHAGE_Entero_IME_EFm5_NC_028826(2)	44.8
L. paracasei T0902						
NODE_1_length_437545_cov_148.551080	incomplete	10	9	386566-391945	PHAGE_Bacill_G_NC_023719(2)	49.2
NODE_3_length_307816_cov_118.004826	incomplete	20	10	242422-251804	PHAGE_Staphy_SPbeta_like_NC_029119(3)	46.37
NODE_4_length_235316_cov_117.084246	incomplete	20	8	201421-209815	PHAGE_Prochl_P_SSM2_NC_006883(3)	49.51

Contig	Completeness	Score	Total Proteins	Region Position	Most Common Phage	GC (%)
NODE_8_length_136053_cov_119.565740	incomplete	60	15	56818-68446	PHAGE_Entero_IME_EFm5_NC_028826(2)	45.5
L. paracasei T1301						
NODE_1_length_437545_cov_144.968924	incomplete	10	9	386566-391945	PHAGE_Bacill_G_NC_023719(2)	49.2
NODE_3_length_307816_cov_129.130097	incomplete	20	10	242422-251804	PHAGE_Staphy_SPbeta_like_NC_029119(3)	46.37
NODE_4_length_235316_cov_125.059548	incomplete	20	8	25502-33896	PHAGE_Prochl_P_SSM2_NC_006883(3)	49.51
NODE_8_length_136053_cov_129.613623	questionable	70	16	66279-80812	PHAGE_Entero_IME_EFm5_NC_028826(2)	44.87
L. paracasei T1304						
NODE_1_length_437546_cov_145.587285	incomplete	10	9	386566-391945	PHAGE_Bacill_G_NC_023719(2)	49.2
NODE_2_length_307625_cov_129.244541	incomplete	20	10	242455-251804	PHAGE_Staphy_SPbeta_like_NC_029119(3)	46.37

Contig	Completeness	Score	Total Proteins	Region Position	Most Common Phage	GC (%)
NODE_3_length_235315_cov_126.925271	incomplete	20	8	25502-33896	PHAGE_Prochl_P_SSM2_NC_006883(3)	49.51
NODE_9_length_136053_cov_134.368528	questionable	70	16	66279-80812	PHAGE_Entero_IME_EFm5_NC_028826(2)	44.87
L. paracasei T1901						
NODE_1_length_437548_cov_148.094406	incomplete	10	9	386568-391947	PHAGE_Bacill_G_NC_023719(2)	49.2
NODE_2_length_307816_cov_114.422400	incomplete	30	18	47496-65362	PHAGE_Staphy_SPbeta_like_NC_029119(3)	46.89
NODE_3_length_235315_cov_118.819834	incomplete	20	8	201420-209814	PHAGE_Prochl_P_SSM2_NC_006883(3)	49.51
NODE_9_length_136053_cov_134.123323	incomplete	60	15	56818-68446 info_outline	PHAGE_Entero_IME_EFm5_NC_028826(2)	45.5
